# Personalized High-Definition Transcranial Direct Current Stimulation for the Treatment of Depression: A Randomized Clinical Trial

**DOI:** 10.1001/jamanetworkopen.2025.31189

**Published:** 2025-09-11

**Authors:** Mayank A. Jog, Viviane Norris, Paloma Pfeiffer, Brandon Taraku, Suzanne Kozikowski, Jacquelyn Schneider, Michael Boucher, Marco Iacoboni, Roger Woods, Katherine Narr

**Affiliations:** 1 Department of Neurology, UCLA, Los Angeles; 2 Department of Psychiatry and Biobehavioral Sciences, UCLA, Los Angeles; 3 Department of Bioengineering, UCLA, Los Angeles

## Abstract

**Importance:**

Transcranial direct current stimulation (tDCS) shows promise for treating depression. The precision and focality of stimulation may influence therapeutic outcomes; thus, investigation of personalized focal high-definition (HD) tDCS for treating depression is needed.

**Objective:**

To determine whether HD-tDCS therapy can improve mood in patients with moderate to severe depression.

**Design, Setting, and Participants:**

This randomized clinical trial used a double-blind, sham-controlled, parallel study design. The trial was conducted from December 1, 2020, to March 7, 2024, at UCLA. Each participant received a 4-week follow-up. Participants were required to meet diagnostic criteria for a current major depressive episode (evaluated using the Mini International Neuropsychiatric Interview), present moderate to severe depressive symptoms (ie, have a 17-item Hamilton Depression Rating Scale [HAMD] score ≥14 and <24), be either treatment naive or receiving a stable standard antidepressant regimen, and be 18 to 65 years of age. Key exclusion criteria included treatment-resistant depression, bipolar disorder, or schizophrenia. Of 560 volunteers screened, 71 were found to be eligible for participation.

**Interventions:**

Participants received 20 minutes of active or sham HD-tDCS each day for 12 consecutive working days. Structural magnetic resonance imaging and frameless stereotaxic neuronavigation were used to personalize the HD-tDCS configuration to each participant.

**Main Outcomes and Measures:**

Pretreatment to posttreatment change in the HAMD score.

**Results:**

Seventy-one participants (44 female [62.0%]; mean [SD] age, 34.3 [10.4] years) were randomized to receive active (n = 40) or sham (n = 31) HD-tDCS therapy. The primary outcome measure, mean (SD) pretreatment to posttreatment change in HAMD score, differed significantly between treatment groups (group difference, −2.2 [4.3]; *P* = .04; Cohen *d*, −0.50 [95% CI, −0.99 to −0.01]). Post hoc 2-sample *t* tests revealed significant decreases in HAMD scores within both groups, with (significantly) greater decreases in the active treatment group (active HD-tDCS group, −7.8 [4.2]; sham HD-tDCS group, −5.6 [4.4]). HD-tDCS was well-tolerated, with mild to no adverse effects. Exploratory analyses indicated a significant active treatment-related improvement in the anxiety dimension of the primary outcome measure (group difference, −0.68 [1.42]; *P* = .049; Cohen *d*, −0.48 [95% CI, −0.96 to −0.004]).

**Conclusions and Relevance:**

In this randomized clinical trial of HD-tDCS in participants with moderate to severe depression, the 12-day HD-tDCS therapy was observed to significantly improve mood with a moderate effect size. Similar effect sizes in pharmacotherapy, psychotherapy, and conventional tDCS–based therapy have been observed to occur substantially later, highlighting the clinical potential of more precise and focal tDCS in depression. Follow-up studies with appropriate maintenance treatments are needed to clarify the persistence of antidepressant effects, as well as possible therapy optimizations. Exploratory analyses indicated that HD-tDCS therapy also may be salient for treating anxiety disorders.

**Trial Registration:**

ClinicalTrials.gov Identifier: NCT04507243

## Key Points

**Question:**

Is high-definition transcranial direct current stimulation (HD-tDCS) a viable treatment for major depression?

**Findings:**

In this randomized clinical trial of 71 participants with moderate to severe depression, statistically significant mood improvement was observed in the group who received active HD-tDCS therapy for 12 consecutive working days, relative to a control group who received sham HD-tDCS therapy.

**Meaning:**

HD-tDCS may provide an effective and fast-acting alternative treatment for depression, pending replication and characterization of long-term effects and neural mechanisms.

## Teaser

This randomized clinical trial investigated personalized high-definition transcranial direct current stimulation therapy to the left dorsolateral prefrontal cortex among patients with moderate to severe depression.

## Introduction

Depression is one of the most common mental health disorders.^1,2^ Standard pharmacotherapies and psychotherapies are only moderately successful; both require several weeks of treatment to achieve remission^3,4^ and show partial response or nonresponse in more than one-third of patients with depression, even after multiple sequenced treatments.^5,6,7^ Pharmacotherapies can also be associated with several undesirable adverse effects, including sexual dysfunction, weight gain, and sleep disturbance.^8,9^ These factors highlight the need to develop therapies that are effective, fast-acting, and less likely to induce adverse effects.^10^

Brain imaging studies have shown that depression is associated with altered neural activity across a wide range of brain regions, resulting from a broader pattern of dysfunction between large-scale brain networks.^11,12,13,14^ Specifically, the extant literature suggests that depressive symptoms result from dysfunction within the emotion-regulating frontoparietal and mediating salience brain networks.^15,16^ This dysfunction is linked with dysregulated processing of emotions and self-referential thoughts, associated with hyperactivity within the limbic and default mode networks, respectively.^17,18,19^

Transcranial direct current stimulation (tDCS) is an emerging safe and noninvasive brain stimulation technique that provides the ability to target dysfunctional networks for treatment. tDCS uses electrodes placed on the scalp to administer tolerable electric currents at brain targets.^20^ The interconnected structure of brain networks means that often perturbation of a single brain location or network node can be sufficient to modulate large-scale networks of interest.^21^ In depression, tDCS studies typically target the dorsolateral prefrontal cortex (DLPFC), a key node in the dysfunctional frontoparietal brain network. Neuroimaging studies of the DLPFC in depression have indicated a left-right asymmetry in neural activity^22,23^; consequently, conventional tDCS configurations place the excitatory tDCS electrode over the hypoactive left DLPFC and the return inhibitory electrode over the hyperactive right DLPFC or the neutral contralateral supraorbital scalp location.^24^ However, conventional configurations have been shown to induce peak current densities in the frontopolar cortex rather than in the targeted DLPFC locations,^25,26^ and this nonspecificity may impact efficacy.

More specific targeting of cortical regions can be achieved using the recently developed high-definition (HD) tDCS configuration.^26,27,28^ Neuroimaging markers indicate that personalized spatially specific left DLPFC HD-tDCS is better at modulating depression-relevant network regions than similarly personalized conventional tDCS.^29,30^ Consequently, we conducted a clinical trial to investigate personalized left DLPFC HD-tDCS therapy for major depression. Here we investigated the trial hypothesis relating to clinical efficacy, that is, whether active HD-tDCS can induce a significantly greater improvement in pretreatment to posttreatment depressed mood compared with sham HD-tDCS.

## Methods

### Study Design

The Imaging-Guided tDCS Therapy in Major Depression study was a randomized, double-blind, sham-controlled clinical trial conducted at UCLA from December 1, 2020, to March 7, 2024. A parallel design was used, and participants were randomized using a computer-generated list to receive active or sham HD-tDCS therapy in a 1:1 ratio while stratifying for sex. The study was approved by the UCLA Institutional Review Board, and all participants provided written or electronically signed informed consent. We followed the 2025 Consolidated Standards of Reporting Trials (CONSORT) guidelines and included all recommended items in this report. The trial protocol is given in Supplement 1.

### Participants

**Figure 1.**
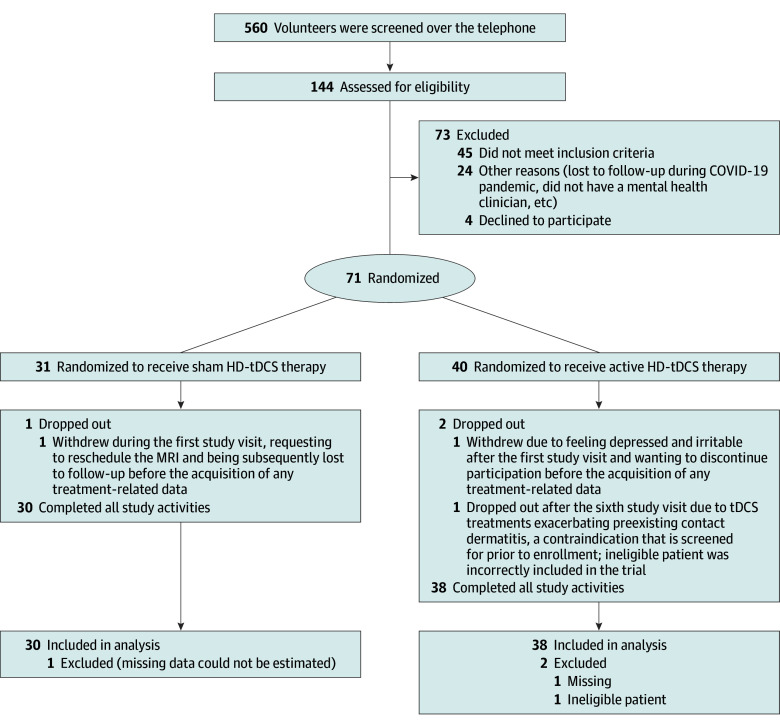
CONSORT Diagram Participants were stratified for sex. HD-tDCS indicates high-definition transcranial direct current stimulation; MRI, magnetic resonance imaging.

As shown in Figure 1, 560 volunteers were screened for participation over the telephone. Of these, 144 passed the screening and were subsequently assessed for eligibility during the initial consultation study visit, which included evaluation by a psychiatrist. For eligibility, participants were required to meet criteria for a current major depressive episode (assessed using the Mini International Neuropsychiatric Interview,^31^ version 7.0.2, and the *Diagnostic and Statistical Manual of Mental Disorders, Fifth Edition*^32^). Participants were also required to be 18 to 65 years of age, have moderate to severe depression (ie, a Hamilton Depression Rating Scale [HAMD]^33^ score ≥14 and <24), and be either treatment naive or receiving a stable standard antidepressant regimen with no change at least 6 weeks prior to and during study participation. Treatment-resistant depression, bipolar disorder, schizophrenia, and neurological disorders were exclusionary (eMethods 1 in Supplement 2 provides detailed inclusion and exclusion criteria). Participants received $50 and parking compensation for each of the 13 in-person visits (ie, the consultation and subsequent 12 treatment visits), and a $20 gift card for participating in assessments conducted at the exploratory 2- and 4-week posttreatment time points.

Overall, 71 participants met eligibility criteria and were randomized to receive active (n = 40) or sham (n = 31) HD-tDCS therapy. Of these, 3 participants discontinued study participation (details in eMethods 2 in Supplement 2). Briefly, one participant from each group dropped out before the acquisition of any treatment-related data. These missing data cannot be estimated from pretreatment data alone; consequently, both participants were excluded from analyses. An additional active HD-tDCS group participant dropped out midway due to worsening of preexisting contact dermatitis. This condition is a contraindication^34^ that all participants were asked about during the initial telephone screening and was specified as exclusionary in the screening script. As recommended by Fergusson et al,^35^ this participant was excluded from analysis. The remaining 68 participants completed all study activities and follow-ups, did not have instances of missing data, and were included in the intention-to-treat analysis.

### Intervention

Each participant received 20 minutes of active or sham HD-tDCS treatment every day for 12 consecutive working days. Participants were seated during treatments and asked to stay awake and let their minds wander without focusing on any specific task or thoughts. Treatments were administered using a double-blind device (Model 5100D; Soterix Medical) operated using participant-specific codes that were assigned according to a computerized list at randomization. Active treatment involved 20 minutes of 2-mA tDCS, including brief (30-second) periods of ramping. Sham treatment involved a ramp up and ramp down, followed by the device outputting 0.065 mA over the rest of the 20-minute session.

The HD-tDCS stimulation configuration consisted of five 2 × 2-cm electrodes, with the central excitatory positive-polarity (ie, anodal) electrode placed over the stimulation target, and the 4 return negative-polarity (ie, cathodal) electrodes placed 5 cm away from the central electrode in a symmetric arrangement. This configuration was personalized to target a specific location in the left DLPFC (x = −46, y = 44, and z = 38 mm in Montreal Neurological Institute coordinate space, based on prior studies^29,36^) using structural magnetic resonance imaging data acquired during the consultation study visit and frameless stereotaxic neuronavigation (eMethods 3 in Supplement 2).

### Data Acquisition

Mood was assessed using the 17-item HAMD scale at the consultation, baseline (visit 1), midtreatment (visit 6), posttreatment (visit 12), and 2- and 4-week posttreatment time points. The HD-tDCS treatments spanned 12 consecutive working days. Treatment-related discomfort was assessed after every administration of HD-tDCS using the modified Generic Assessment of Side Effects (GASE^37^) survey, similar to the method used by Jog et al.^29^ The Adverse Events Questionnaire (AEQ) was also administered at the posttreatment visit per recommendation.^38^ Finally, both participants and assessors were asked to guess treatment allocation (active or sham) at the posttreatment time point to evaluate blinding integrity. The 2- and 4-week posttreatment time points were exploratory additions to the study protocol, in light of studies published at the time our trial was initiated, indicating that the clinical effects of tDCS may be delayed.^39,40,41^ Unblinding was performed after all data acquisition had been completed. Neuroimaging data not pertinent to the hypotheses being investigated in this study were also acquired during the trial and will be reported separately.

### Outcomes

The primary outcome measure was mood improvement, defined as the pretreatment to posttreatment change in HAMD scores. Secondary outcome measures included alternative measures of mood improvement, including percentage change in HAMD score during the same time period, and rates of posttreatment response (at least 50% pretreatment to posttreatment change) and remission (posttreatment HAMD score ≤7). Exploratory analyses investigated changes in previously identified^42^ symptom dimensions of the HAMD (including anxiety, depression, and somatic and insomnia symptoms) and the overall trajectory of mood improvement during the course of the trial.

### Statistical Analysis

All statistical analyses used a significance threshold of 2-tailed *P* < .05. Analysese were conducted using MATLAB, version R2022a (The MathWorks Inc).

#### Primary Hypothesis

We hypothesized that pretreatment to posttreatment changes in mood would differ significantly between the active and sham treatment groups. A 2-sample *t* test was used to compare the pretreatment to posttreatment change in HAMD scores between the 2 groups. Pretreatment scores were calculated as the mean of the scores measured at the consultation and the baseline study visits; these scores did not differ significantly between these pretreatment time points for either treatment group (eMethods 4 in Supplement 2).

#### Sample Size

We planned to recruit 50 participants per treatment group, to ensure 80% power to detect group differences at a significance level of *P* = .05 (2-tailed) for an effect size of 0.57. This effect size was estimated by calculating the mean of the pooled estimates of effect sizes reported in meta-analyses that investigated antidepressant effects of left DLPFC tDCS^43,44^ (albeit the studies included in both meta-analyses used different stimulation configurations, current intensities, and dose duration). Due to enrollment challenges during the COVID-19 pandemic, the planned sample sizes were not achieved by the end of the recruitment period; consequently, group sizes were somewhat unbalanced, and statistical power was duly affected.

#### Additional Analyses

Group differences in secondary outcomes were investigated using the 2-sample *t* test for the percentage of change measure and the χ^2^ test for the posttreatment response and remission rate measures. Two-sample *t* tests were also used to investigate group differences in treatment-related discomfort, as well as the previously described exploratory analyses.

Differences in clinical and demographic characteristics between the 2 groups were investigated using the 2-sample *t* and χ^2^ tests for continuous and categorical variables, respectively. The χ^2^ test was also used to investigate group differences in guesses of the type of treatment received (active or sham) by participants and assessors, to evaluate blinding integrity.

## Results

Seventy-one participants (44 female [62.0%] and 27 male [38.0%]; mean [SD] age, 34.3 [10.4] years) were randomized to receive active (n = 40) or sham (n = 31) HD-tDCS therapy. Clinical or demographic characteristics did not differ significantly between the 2 groups (Table 1). Overall treatment-related discomfort measured using the GASE survey and AEQ also did not differ significantly between the 2 groups (eResults 1 in Supplement 2). Some individual adverse effects on the GASE survey (rash or itching mean [SD] scores, 0.18 [0.40] vs 0.01 [0.06]; *P* = .02) and adverse event items on the AEQ (skin redness mean [SD] score, 1.32 [0.51] vs 1.04 [0.16] [*P* = .006]; burning sensation mean [SD] score, 1.33 [0.51] vs 1.08 [0.37] [*P* = .03]) did show statistically significant differences (uncorrected for multiple comparisons); however, group differences were minimal (eResults 1 in Supplement 2). Finally, no significant group differences were observed in guesses of active and sham treatment allocation by participants or assessors (participants: χ^2^ = 1.03 [*P* = .31]; assessors: χ^2^ = 0.28 [*P* = .60]; group-wise guesses of active treatment by participants were 23 active HD-tDCS and 16 sham HD-tDCS; guesses by assessors, 18 active HD-tDCS and 13 sham HD-tDCS).

**Table 1.** Clinical and Demographic Characteristics of Participants

Characteristic	Treatment group		
	Total (n = 71)	Active HD-tDCS (n = 40)	Sham HD-tDCS (n = 31)
Demographic			
Sex, No. (%)			
Female	44 (62.0)	23 (57.5)	21 (67.7)
Male	27 (38.0)	17 (42.5)	10 (32.3)
Age, mean (SD), y	34.3 (10.4)	36.0 (10.1)	32.3 (10.6)
Duration of education, mean (SD), y	15.2 (2.4)	15.2 (2.2)	15.1 (2.6)
BMI, mean (SD)	27.0 (6.2)	27.3 (6.7)	27.0 (5.6)
Clinical			
Pretreatment HAMD scores, mean (SD)^a^	17.7 (2.6)	17.7 (2.5)	17.8 (2.7)
Onset age, mean (SD), y	23.3 (9.1)	24.6 (10.2)	21.6 (7.1)
Duration of illness, mean (SD), y^b^	1.6 (4.3)	1.3 (1.7)	1.9 (6.1)
No. of depressive episodes, mean (SD)^c^	7.1 (9.9)	7.6 (10.6)	6.7 (9.7)
No. of participants			
Unipolar depression	70	39	31
Bipolar (type II) depression	1	1	0
SSRI, SNRI, or NDRI treatment	50	30	20
Treatment naive	18	9	9

Abbreviations: BMI, body mass index (calculated as the weight in kilograms divided by the height in meters squared); HAMD, Hamilton Depression Rating Scale; HD-tDCS, high-definition transcranial direct current stimulation; NDRI, norepinephrine-dopamine reuptake inhibitor; SNRI, serotonin-norepinephrine reuptake inhibitor; SSRI, selective serotonin reuptake inhibitor.

^a^ Scores ranging 14 to less than 24 indicate moderate to severe depression.

^b^ Includes 68 participants.

^c^ Includes 27 participants.

### Primary Outcome

Pretreatment to posttreatment changes in HAMD scores were significantly different between the active and sham treatment groups (mean [SD] group difference, −2.2 [4.3]; *P* = .04; Cohen *d*, −0.50 [95% CI, −0.99 to −0.01]). Post hoc 2-sample *t* tests revealed significant HAMD score decreases over time within both groups, with (significantly) greater decreases in the active treatment group (−7.8 [4.2] vs −5.6 [4.4]) (Table 2).

**Table 2.** Primary and Secondary Outcomes

Outcome	Treatment group		Effect size (95% CI)	*P* value
	Active HD-tDCS (n = 38)	Sham HD-tDCS (n = 30)		
Primary				
Change in HAMD score, mean (SD)	−7.8 (4.2)	−5.6 (4.4)	−0.50 (−0.99 to −0.01)^a^	.04
Secondary				
Change in HAMD score, mean (SD), %	−44.6 (23.9)	−31.3 (23.5)	−0.55 (−1.04 to −0.06)^a^	.02
Response, No. (%)	16 (42.1)	8 (26.7)	2.00 (0.71 to 5.63)^b^	.19
Remission, No. (%)	15 (39.5)	4 (13.3)	4.24 (1.23 to 14.61)^b^	.02

Abbreviations: HAMD, Hamilton Depression Rating Scale; HD-tDCS, high-definition transcranial direct current stimulation.

^a^ The effect size is Cohen *d.*

^b^ The effect size is odds ratio.

### Secondary Outcomes

Mean (SD) pretreatment to posttreatment percentage changes in HAMD scores were also significantly different between the treatment groups (group difference, −13.4% [24.4%]; *P* = .02; Cohen *d*, −0.55 [95% CI, −1.04 to −0.06]). Post hoc 2-sample *t* tests showed that the percentage changes over time within both groups were significant and negative, with a (significantly) larger magnitude change in the active treatment group (−44.6% [23.9%] vs −31.3% [23.5%]). Posttreatment response rates did not differ significantly between the 2 treatment groups (*χ^2^* = 1.75 [*P* = .19]; responders in the active group, 16 of 38 [42.1%]; responders in the sham group, 8 of 30 [26.7%]). Posttreatment remission rates were observed to differ significantly, with greater remission in the active treatment group (*χ^2^* = 5.69 [*P* = .02]; remission in the active group, 15 of 38 [39.5%]; remission in the sham group, 4 of 30 [13.3%]).

### Exploratory Analyses

Mean (SD) pretreatment to posttreatment changes in the anxiety symptom dimension within the HAMD also differed significantly between the 2 treatment groups (group difference, −0.68 [1.42]; *P* = .049; Cohen *d*, −0.48 [95% CI, −0.96 to −0.004]), driven by significant active treatment-related improvements in both psychic and somatic anxiety (eResults 2 in Supplement 2). No individual HAMD items worsened with active treatment; in fact, 16 of 17 items significantly improved (eResults 3 in Supplement 2).

**Figure 2:**
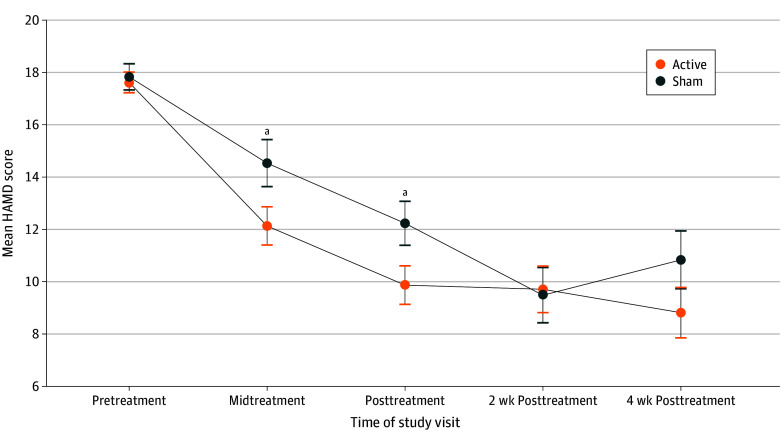
Trajectory of Mood Changes The plot shows the mean 17-item Hamilton Depression Rating Scale (HAMD) scores measured at each time point for each treatment group. High-definition transcranial direct current stimulation was administered for 12 consecutive working days. The midtreatment visit corresponds to treatment visit 6; posttreatment visit, treatment visit 12. The HAMD scores reflect depression severity, and a decrease in the HAMD scores over time indicates mood improvement. Error bars indicate SE. ^a^*P* < .05 for group differences in mood improvement.

Finally, we explored the trajectory of HAMD score changes over time (Figure 2 and spaghetti plots in eFigure in Supplement 2). Mean (SD) longitudinal changes in HAMD scores significantly differed between the 2 treatment groups at the midtreatment time point (group difference, −2.2 [4.1]; *P* = .047; Cohen *d*, −0.54 [95% CI, −1.03 to −0.05]), driven by significantly greater score decreases within the active group (HAMD score changes over time, −5.5 [3.7] vs −3.3 [4.3]). Group differences were not significant at the remaining 2- and 4-week posttreatment time points (eTable in Supplement 2).

## Discussion

In this randomized, double-blind, sham-controlled clinical trial, we evaluated the clinical effects of personalized left DLPFC HD-tDCS therapy. Participants with moderate to severe depression were randomized to receive 12 sessions of active or sham HD-tDCS treatment, and despite the presence of placebo effects, significant mood improvement and remission were observed in the active group relative to the sham group. Blinding integrity was verified, treatments were well tolerated with mild to no adverse effects, and there were no significant pretreatment differences in clinical or demographic characteristics between the 2 groups. Overall, our findings demonstrate the clinical potential of personalized HD-tDCS as a safe and fast-acting antidepressant therapy for moderate to severe depression.

### Key Innovation: the HD Configuration

Previous studies of tDCS in depression have used conventional stimulation configurations. These typically consist of 2 large (5 × 7-cm) electrodes, with the stimulating electrode positioned over the left DLPFC brain target, and the return electrode placed over a pertinent location in the contralateral hemisphere.^24,39,45,46,47,48^ State-of-the-art computational models have shown that the large size of the electrodes results in diffuse, nonfocal stimulation of the left DLPFC,^26^ and their bilateral placement induces current-density hotspots in regions other than the intended DLPFC target.^25,49^

In contrast, the 4 × 1 HD-tDCS configuration used in the present trial has been shown to result in more focal and specific stimulation of the targeted brain region.^26,27,28^ Further, prior neuroimaging work performed by several of our investigators indicated that spatially specific HD-tDCS may be more optimal for targeting depression-relevant network dysfunction.^29,30,48^ Here, an HD and a conventional configuration were personalized to target the same left DLPFC coordinate as used in this study. Empirical in vivo measurements confirmed accurate electrode placement and comparable dose-delivery at the left DLPFC and showed enhanced structural and functional modulation of depression-relevant network regions by HD-tDCS.^29,30,50^

In the present study, active HD-tDCS significantly improved mood compared with sham HD-tDCS with a moderate effect size (Cohen *d*, −0.50) after 12 days of therapy. Exploratory analyses suggest that significant improvements of a comparable effect size may occur as early as the midtreatment time point (Cohen *d*, −0.54). In contrast, previous large-sample clinical trials evaluating conventional tDCS configurations in depression have either shown no effects^51^ or smaller or comparable effect sizes at substantially later time points (ELECT-TDCS [Escitalopram vs Electrical Current Therapy for Treating Depression Clinical Study]^39^ week 8 Cohen *d*, <0.43; EMPOWER^45^ week 4 Cohen *d*, <0.37). The substantial delay in clinical effects could result from less network modulation for the reasons discussed earlier. Notably, all of the listed trials used higher doses (higher current intensities [2.5 mA^51^ vs 2 mA]), longer session durations (30 minutes^39,45,51^ vs 20 minutes), and a greater number of sessions (≥15^39,45,51^ vs 12). Overall, simulations, neuroimaging markers, and clinical effects indicate that spatially specific HD-tDCS may be more optimal than conventional tDCS for treating depression. However, head-to-head trials are needed for confirmation.

Mood improvement in the active treatment group relative to the sham group did not exceed the minimal clinically important difference threshold defined by Hengartner and Plöderl.^52^ Pharmacotherapies have also consistently failed to exceed the minimal clinically important difference threshold in the acute treatment phase,^52,53,54^ and longer follow-up intervals were required to determine clinical relevance. Even so, observed effects compared favorably with those of established therapies. Meta-analyses indicated a small to moderate effect size for pharmacotherapies and psychotherapies after 6 weeks of treatment,^55,56^ with the former often being associated with undesirable adverse effects. In contrast, HD therapy appeared faster acting, showing a moderate effect size earlier with mild to no adverse effects. While indirect, these comparisons indicate the clinical viability of HD-tDCS therapy for depression.

### Potential for Treating Anxiety

Anxiety disorders can significantly impair daily life, are highly prevalent, and often co-occur with other mental health disorders, including depression.^57^ Biologically, anxiety symptoms have been shown to consistently associate with disruptions in the DLPFC-insula-amygdala network circuitry.^14,58^ Exploratory analyses in the present study showed that active left DLPFC HD-tDCS therapy significantly improved anxiety symptoms (derived using robust structures identified by independent factor analyses^42^), relative to sham therapy. Follow-up tests revealed significant improvements in both psychic and somatic components of the anxiety measure. These observations provide preliminary evidence for investigating left DLPFC HD-tDCS therapy for anxiety.

### Safety and Tolerability

Dose parameters used in this study were well within the tDCS safety thresholds.^59,60^ Administered treatments were well-tolerated, and similar to prior studies,^39,45^ only mild to no adverse effects were observed. Active HD-tDCS also did not significantly worsen any individual HAMD item (in fact, 16 of 17 items significantly improved [uncorrected for multiple comparisons]).

### Limitations 

Our study has several limitations. The present study was not designed to investigate interactions between HD-tDCS and psychotropic medications. Observed therapeutic effects may have been influenced by participants’ medication status, and appropriately designed trials are needed to clarify possible interactions of HD-tDCS with psychotropic medications. Treatment effects may also have been influenced by the 0.065-mA sham HD-tDCS current that may not be physiologically inert^61^; future trials should consider using sham conditions that involve zero currents outside of ramping.

The study was also not designed to empirically validate personalization. Well-established computational models^62,63^ support the need for personalization in our application (eMethods 3 in Supplement 2); nevertheless, validation studies that include nonpersonalized control groups are needed.

Our study protocol did not include maintenance treatment sessions during the exploratory follow-up period, which may have contributed to the nonsignificant findings at the 2- and 4-week posttreatment time points. However, depressive symptoms during this period did appear to remain stable in the active group while increasing in the sham group. Future trials with longer follow-up intervals and incorporating maintenance treatments are needed to clarify the long-term longitudinal effects of HD-tDCS therapy.

HD-tDCS therapy parameters may also benefit from further optimization. In the present study, HD-tDCS therapy only included 12 treatment sessions, based on previous studies of conventional tDCS in depression.^24^ However, mood scores in the trial continued to improve until the final administered treatment (ie, the posttreatment time point in Figure 2), indicating that adding more treatment sessions may further improve efficacy. Additionally, participants in the study were administered HD-tDCS at rest. The effects of tDCS are also known to depend on brain state,^64^ such that clinical effects may improve with priming of a particular functional brain network before or during tDCS. Complementary targets (eg, right DLPFC^24^) and alternative personalization strategies based on functional neuroimaging and insights from the neural substrate of HD-tDCS are also important factors that need to be considered. In vivo imaging approaches that enable comparison of network engagement between different parameter choices^29,65^ could be key in efficiently optimizing HD-tDCS therapy for depression.

## Conclusions

In this randomized clinical trial, mood in participants with moderate to severe depression significantly improved after 12 days of left DLPFC active HD-tDCS therapy relative to sham treatment. Future studies incorporating appropriate maintenance treatments are needed to clarify the persistence of mood improvement, as well as whether antidepressant effects could be further improved by optimizing therapy parameters. Exploratory analyses indicated that left DLPFC HD-tDCS may also be viable for treating anxiety symptoms.

## Notes

Supplement 1.
Trial Protocol

Supplement 2. **eMethods 1.** Inclusion and Exclusion Criteria **eMethods 2.** Trial Dropouts **eMethods 3.** Personalization Methodology **eMethods 4.** HAMD Scores at Consultation and Baseline **eResults 1.** Treatment-Related Discomfort **eResults 2.** Treatment-Related Changes in HAMD Symptom Dimensions **eResults 3.** Treatment-Related Changes in Individual HAMD Items **eFigure.** Spaghetti Plots of HAMD Scores Over Time **eTable.** Outcome Measures Over Time
eReferences

Supplement 3.
Data Sharing Statement
