# Supplementary material for: Personalized High-Definition Transcranial Direct Current Stimulation for the Treatment of Depression: A Randomized Clinical Trial
Source: JAMA Netw Open. 2025 Sep 11;8(9):e2531189. doi: 10.1001/jamanetworkopen.2025.31189 (PMC12426800; doi:10.1001/jamanetworkopen.2025.31189)
Supplement: Supplement 1. — Trial Protocol [file jamanetwopen-e2531189-s001.pdf]

Trial Protocol and Statistical Analysis Plan:

The current work examines whether personalized transcranial direct current stimulation (tDCS) therapy improves depressed mood. The analyzed data is from the R33 phase (only) of a 2-phase NIH trial (R61/R33MH110526). The primary outcome measure of the R33 phase of the trial (NCT04507243) assesses the joint longitudinal relationship between change in mood and change in regional cerebral blood flow in active compared to sham tDCS conditions. The current work evaluates the first part of this outcome measure, i.e., change in mood. Notably, the R33 phase was designed to investigate changes in mood; power-calculations justifying the sample-size and the statistical test for investigating tDCS-related mood improvement were both specified *a priori* in the (approved) R61/R33 grant.

Please find attached a brief overview of the R61/R33 trial to provide context (below), followed by the IRB-approved R33 phase trial protocol (starting on the next page), and the statistical analysis plan as specified in the awarded grant (last page of this document, with sample-size justification and statistical test highlighted in yellow and green respectively). The full, approved R61/R33 grant documentation can also be provided if needed.

A brief overview of the R61/R33 MH110526 trial:

Both the R61 and R33 phases of the trial were submitted to NIH as a single submission in 2017, and the grant was approved on 07/28/2017. The R61 phase of the trial was completed in 2020, and findings (a) confirmed targeting accuracy (PMID: 34555822), and (b) demonstrated target engagement (functional plasticity (PMID: 33627624), structural plasticity (PMID: 36801903)) of the personalized tDCS configuration being investigated in this R33 phase trial. After meeting the Go/No Go criteria for the R61 phase, the R33 phase research was approved for funding on 07/31/2020, based on NIMH administrative review with no additional peer review. Our request for administrative approval to proceed to the R33 phase did not propose any significant deviations from the R33 as described in the original R61/R33 submission[1]. The timeline of the R33 phase substantially overlapped with the COVID pandemic, and the trial ultimately had to be discontinued before reaching the target sample size of 100 due to exhaustion of available NIH funds, leading to a smaller than planned sample-size and somewhat unbalanced groups.

[1] The sole change in the protocol was in response to interim publications (e.g. PMID: 28657871) that indicated that tDCS may take longer to have full benefit in depression than anticipated at the time of the R61/R33 proposal. Consequently, two exploratory follow-up timepoints for mood assessments were added to the protocol. This decision was made in advance of enrollment of the first participant, and the additional assessments took place after all of the originally proposed data for a given subject had been fully acquired, with a temporal delay in unblinding being the only trivial deviation from the original protocol.

tDCS STUDY PROTOCOL
Version 04/04/23

In accordance with the NIH/NIMH R61/R33 funding mechanism, which will sponsor this research, this exploratory clinical trial project is divided into two separate phases. The initial R61 Phase (IRB#17-000779) is designed to establish if tDCS engages particular neural circuits, and to determine which tDCS parameters (using standard or HD tDCS) are optimal for neural engagement. The design of the second R33 Phase of the project is based on the outcomes of the R61 Phase (IRB#17-000779), which show that transcranial direct current stimulation (tDCS) applied to the left dorsolateral prefrontal cortex (DLPFC) is able to modulate clinically-relevant neural circuits in patients with major depression. Using novel in-vivo tDCS-MRI methods, our results also demonstrate that high-definition (HD) tDCS is more focal and leads to more prominent changes in both mood-regulating DLPFC and dorsal anterior cingulate (dACC) target regions than conventional tDCS. Using HD tDCS, the R33 will define relationships between target engagement and change in mood and behavior with repeated tDCS in a larger cohort of depressed subjects.

R33 Phase

Sample: Our sample will consist of **100 (R33 phase)** people currently experiencing a major depressive episode (male and female, 18-65 years of age), who are recruited to receive tDCS. Our sample will include subjects meeting DSM-5 criteria for a moderate-to-severe MDE, with Hamilton Depression Rating Scale symptom scores of ≥ 14 and <24. Exclusion criteria include the following: non-English speaking; Schizophrenia Axis I disorder; primary anxiety disorder; Bipolar I disorder and psychotic disorders; any neurological condition or major illness, including seizure disorder; diagnosis of dementia of any type; co-morbid substance abuse in the last three months; contraindications to MR scanning (including pregnancy); contraindications to tDCS (e.g., skin disease or treatment causing irritation); treatment-resistant depression, with a history of a major depressive episode lasting > 2 years or failure to 2 or more antidepressant trials in the current episode; any neuromodulation therapy (e.g., ECT, rTMS, DBS, VNS, or tDCS) within the last 3 months; and active suicidality.

Subjects who are receiving standard antidepressants (SSRIs, SNRIs, TCAs, MAOIs) and antipsychotics are eligible to participate, though must have remained stable for ≥ 6 weeks prior to the first research assessment and remain stable during participation; subjects who are treatment naïve are eligible to participate. Current or past (within the last 1-month) use of anticonvulsants, lithium, psychostimulants, or dexamphetamine is exclusionary. Participants will withhold benzodiazepines, which can impact cortical excitability, for the duration of the trial. We will exclude participants with medical conditions that render the participant high-risk for COVID-19 infection (e.g. diabetes, heart and lung disease). Current use of decongestants or other medication previously shown to interfere with cortical excitability is exclusionary. Subjects who are currently receiving any form of Cognitive Behavioral Therapy, Dialectical Behavioral Therapy, or Acceptance and Commitment Therapy are not eligible. Participants must also be local, meaning they are within commuting distance of UCLA.

We will recruit participants who have completed screening for other UCLA Depression Grand Challenge projects for whom we have current information about symptom severity. Studies include: IRBs #15-001173, 15-000856, 16-001568, 16-001781, 16-000232, 16-001395, and 17-001365. We will also recruit subjects from Outpatient Psychiatry Clinics in the UCLA Health System, UCLA Ambulatory Primary Care practices, and community providers through the use of print and online flyers. Additionally, we will post information about the study and eligibility requirements on our website. Banner ads directing participants to our website will be purchased through Google AdWords and Reddit Ads. We anticipate screening and enrolling a larger number of individuals (approximately 250) in order to meet our target number of completers (100). Subjects will also be recruited by contacting the study team via ClinicalTrials.gov or through ClinicalConnection.com, which provides information about available clinical trials to a wide network of potential study participants. Once their participation in the study is

complete, subjects will be offered the opportunity to participate in another study (IRB # 22-001367). They will be told about this study verbally and flyers with more information will be available to them.

Study Overview: In the R33 phase, subjects will be randomly assigned to HD-tDCS (focal left DLPFC tDCS) or sham tDCS, stratifying for sex (see Table 1). Subjects assigned to the sham condition will not receive active stimulation. Random assignment will be computer-generated and stratified to ensure equal numbers of males and females in each condition.

At the end of the trial, subjects assigned to the sham condition will be offered open label active tDCS for the same duration of subjects randomized to the active conditions.

Table 1. RANDOMIZED GROUPS

**VISIT**	**R33 N**
HD-tDCS (Focal stimulation of left DLPFC tDCS)	50
Sham HD-tDCS	50

See Table 2 for an overview of our longitudinal study design. The study lasts no longer than two months, per subject (tDCS treatment over 2-3 weeks, and a remote assessment of symptoms at 2- and 4-weeks post treatment). Participants may receive study visit reminders via text. The study design includes 1) a remote screening appointment conducted via secure video conference with a consent interview, screening assessments (listed below), and clinical interviews (**Visit #1a**), followed by a *pre-trial* 30-minute structural MRI scan and computerized tests of memory and thinking which will occur on a separate day up to 1-week after the consent and screening assessments(**Visit #1b**); 2) a baseline assessment session, occurring approximately 1-week after the screening appointment, with mood assessments, surveys, computerized tasks (if not already completed during Visit #1b), and a 1-hour functional MRI scan during which tDCS is delivered for about 30 minutes and two tasks probing cognitive control and emotion negativity bias are administered (**Visit #2**); 3) ten single tDCS sessions, occurring on consecutive days excluding weekends, administered outside of the scanner for 20-minutes each (**Visits #3-12**) with tDCS-related side effect ratings at each tDCS session; plus 4) additional mood assessments at **Visit #7**; and 5) a final *post-trial* assessment session that again includes a functional MRI scan with the delivery of tDCS and administration of two functional tasks, followed by a final assessment of mood and cognition (**Visit #13**). In addition, two weeks and four weeks after the post-trial assessment, participants will be asked to complete a mood assessment via phone. During visits #2, #7, and #13, except for the MRI scans, data be will be collected while the participant is in a separate room but speaking via secure video conference (e.g., Zoom) with the research coordinator in a different room. Cognitive tasks may be collected if feasible with social distancing. tDCS sessions will be conducted while the participant is in a separate room and being monitored via secure video conference by the research coordinator from a different room. As such, subjects will receive **tDCS stimulation daily for no more than three weeks** and complete **three comprehensive cognitive assessments (baseline, mid-trial at Visit #7, and after the tDCS series at Visit #13).** Subjects will receive **three MRI scans (pre-trial (Visit #1a), baseline (Visit #2) and post-trial (Visit #13))**. Mood will be monitored throughout the study, at Visits #1, #2, #7 and #13, to monitor response and additionally at 2 and 4 weeks after the post-trial assessment over the phone.

Table 2. SUMMARY OF RESEARCH APPOINTMENTS

VISIT #	Pre-trial 1a & 1b	Baseline 2	3	4	5	6	Mid-trial 7	8	9	10	11	12	Post-trial 13	2 and 4 weeks post trial
Consent	X													
HIPAA	X													
Consult	X													
tDCS only			X	X	X	X	X	X	X	X	X	X		
Brain Scan only	X													
Brain Scan with tDCS		X												
Brain Scan with fMRI tasks		X											X	
Mood Scales	X	X					X						X	X
Cognitive tests	X	X											X	
Time (hours)	5	4	1	1	1	1	3.5	1	1	1	1	1	4	0.5 each

* tDCS sessions will be administered daily for 12 business days.

Procedure*:*
***Visit #1a (Pre-trial screening)*:** In addition to obtaining consent and obtaining HIPAA authorization for research form, the first assessment lasting 3-4 hours will include remote screening to ensure that subjects are eligible and there are no contraindications to either tDCS or scanning. During this initial interview, a PhD-level study member will describe the research study, ensure study inclusion/exclusion criteria are met, and review the informed consent form with the patient. The consent process will be administered via DocuSign, or through a secure database (e.g. REDCap). If administration of consent through either DocuSign or the secure database is not possible, the consent form will be mailed or emailed to the participant (via secure Mednet email) and a PhD-level study member will review the consent form with the participant in detail via secure video conference or phone. If the consent form is emailed, the participant will print a paper copy of the consent form upon receipt. After reviewing the consent form with thePhD-level study member , the participant will sign the consent document, take a picture of the signed page with their phone, and email this scanned copy to the coordinator, in accordance with FDA guidance. The scanned copy of the signature page will be stored on a secure, firewall-protected server at the Brain Mapping Center or in internal REDCap. The participant will provide the signed hard-copy consent at the next in-person visit.
Using secure video conference, a trained clinical rater will conduct a diagnostic interview following the MINI 7.0.2 for DSM-V to ensure diagnoses meet inclusion/exclusion criteria and systematically assess psychiatric clinical symptoms (including current substance use). Through this interview, information about the patient's diagnostic and medication history will be obtained, including age of first depressive episode, number of previous episodes, and duration of current episode.

***Visit #1b (Pre-trial scan)*:** If the research participant passes MRI safety screening, they will then be invited to come to UCLA to complete a 30-minute pre-trial structural MRI scan and some computerized

tests of memory and thinking. The study physician will oversee screening sessions and review eligibility prior to the baseline assessment at Visit 2.
Visit #2 (Baseline assessment): If study inclusion/exclusion criteria are confirmed, subjects will return to the laboratory for comprehensive assessment lasting up to 4 hours, during which subjects complete a battery of neurocognitive tests (if feasible with social distancing, and if not already completed at Visit #1b; see **Assessment Battery** below) and collection of data concerning demographics, health, and handedness. Subjects will be escorted to the Brain Mapping Center. The reconstructed structural MRI data collected at Visit 1b for each subject will be used to guide the placement of the tDCS electrodes in a private room. Once the subject is prepared for tDCS administration, the subject will receive a 1-hour functional imaging scan, broken into separate smaller acquisition blocks, with tDCS delivered for a period of about 30 minutes. During this scan, two functional imaging tasks probing cognitive control and emotion negativity bias are also administered. Except for the MRI scan and electrode placement, data be will be collected while the participant is in a separate room but speaking via Zoom with the research coordinator in a different room.

Visits #3-12**:** For the following ten visits, subjects will return to receive a 20-minute tDCS outside the scanner and complete a side-effects assessment in a private room (see **tDCS procedure** below), with breaks on the weekend. tDCS sessions will be conducted while the participant is in a separate room but being monitored via Zoom by the research coordinator from a different room.

Visit #7**:** Halfway through the trial, participants will complete a shortened assessment battery on the day of their sixth tDCS session (see **Assessment Battery** below). Data be will be collected while the participant is in a separate room but speaking via Zoom with the research coordinator in a different room.

Visit #13**:** At the final tDCS session, participants will complete a shortened assessment battery as on the day of their sixth tDCS, including a single one-hour scan, again with tDCS delivered and two functional imaging tasks administered in the scanner (see **Assessment Battery** below). Except for the MRI scan, data be will be collected while the participant is in a separate room but speaking via Zoom with the research coordinator in a different room.

In addition, two and four weeks after the post-trial assessment, participants will be asked to complete mood assessments via phone, lasting approximately 30 minutes each.

Subjects will be compensated for their participation in the study and receive $50 for each of the 13 in-person research visits, and up to a $25 e-gift card for completing both mood assessment scales post-trial, for a total of up to $725. Payment will be provided after completion of the first research visit (Visit #1b), final research visit (Visit #13), and again after the final mood assessment conducted over the phone 4 weeks post-trial. Parking at UCLA will be reimbursed for each of the 13 research visits with reimbursement provided at the time of each visit. If circumstances (e.g., car breakdown) arise rendering subjects unable to travel to their tDCS sessions, ride-share (e.g., Uber, Lyft) fares will be reimbursed up to $50 each for a maximum of 10 sessions, for a maximum of $500. Documentation of circumstances that require ride-share compensation may be required.

tDCS procedure: Transcranial direct current stimulation (tDCS) is a non-invasive method of neuromodulation that uses electrodes placed on the scalp to deliver a constant, low current. TDCS has been used as an experimental treatment for major depression. Study investigators are trained to administer tDCS. In this study, subjects will receive concurrent MRI-tDCS at baseline (Visit #2), followed by 10 daily tDCS sessions each lasting 20 minutes (Visits #3-12), and ending with a final concurrent MRI-tDCS session (Visit #13). tDCS will be administered using Neuroconn (www.neuroconn.de and Soterix (http://soterixmedical.com) MR compatible systems and software control of stimulation waveforms enable double-blind experimental design. In the R61 Phase (IRB#17-000779), two electrode configurations were applied: 1) C-tDCS using 7 cm x 5 cm rectangular pad electrodes or 2) HD-tDCS using 4x1 ring electrodes (radius: 5 cm) by Soterix (http://soterixmedical.com). For Sham, current was

ramped up/down for 20 secs via Neuroconn tDCS software control. In general, tDCS does not produce scalp sensations except while current is being adjusted; transient stimulation mimics the effects of active stimulation and minimizes placebo effects, arousal, attention, and other active tDCS effects.

In the R33 Phase, only the HD-tDCS electrode configuration will be applied as described above. For Sham, current will again ramp up/down for 20 secs via Neuroconn tDCS software control.

Monitoring: To foster continued safety and participation of subjects, the PIs or qualified research staff will be in daily contact with enrolled participants. All enrolled subjects are required to be under the simultaneous care of a primary care provider. Subjects enrolled in the tDCS trial will thus also receive monitoring by their treating or referring physician for the entire length of their participation.

Assessment Battery: The following types of data will be collected at times indicated in Table 2. See Table 3 for an overview of measures to be collected. Research staff will collect measures electronically.

Clinical Assessments: The Hamilton Depression Rating Scale (HAMD, 17 item) [1] supplemented with IDS-C [2] will serve as the primary outcome measure of clinical response. This scale will be used to establish therapeutic response and to test for relationships with imaging measures. Additional assessments will include the Quick Inventory of Depressive Symptomatology Self-report (QIDS-SR) [2]. Rumination styles will be assessed using two measures, the Ruminative Response Scale of the Response Styles Questionnaire [4] and the Rumination-Reappraisal subscale of the Thought Control Questionnaire [5]. Assessment of adverse effects may be assessed using the General Assessment of Side-effects (GASE) form, doctor version.

The above clinical scales will be supplemented by questionnaires that enable the assessment of anxiety symptoms (DASS-21 anxiety and stress subscale) [9,10].

Neurocognitive Tasks: Comprehensive assessments sessions will include a standardized neuropsychological assessment battery, supplemented by computerized tasks to assess memory and executive function [6]. Neurocognitive tasks will be administered if feasible with social distancing.

Imaging: Noninvasive neuroimaging will occur on a Siemens 3T system in close proximity to our research testing spaces. Subjects will complete 1) high-resolution T1-and T2-weighted structural scans using Human Connectome Lifespan project protocols (www.humanconnectome.org (Visit #1b); 2) a 3D GRASE pCASL sequence to measure regional changes in rCBF; 3) a magnetic field mapping scan to map tDCS-induced magnetic field; and 4) two brain activation tasks that probe cognitive control/response inhibition and negative emotion bias (Visits #2 and 13).

Table 3. Assessment battery

Domain	Measure(s)
**Diagnosis & Clinical History**	Clinical interview to ensure subjects meet DSM-5 inclusion/exclusion criteria; history of illness course, treatment, and medication use. Current health (e.g., Medical comorbidities; MRI screening form) and handedness.
**Demographics**	Standard measures of demographics (age, sex, ethnicity, education, etc.)
**Symptom & Mood Scales**	Standard measures of current symptom severity and therapeutic response (e.g., HAMD, QIDS-SR,); measures of suicide symptom severity; side-effects assessment (e.g. GASE)

	Supplementary measures that enable assessment of anxiety
	Symptoms of rumination (e.g., Ruminative Response Scale of the Response Styles Questionnaire; Rumination-Reappraisal subscale of the Thought Control Questionnaire)
**Neurocognitive Function (administered if feasible with social distancing)**	Standardized neuropsychological battery to assess memory and executive function (e.g., NIH toolbox cognitive assessments), emotion recognition (e.g., Penn Emotion Recognition Test)
**Neuroimaging**	T1- and T2-weighted structural scans; 3D GRASE pCASL scan; magnetic field mapping scan; fMRI scans (Affective Go/NoGo and Face-Matching tasks) in R33 phase only

References

1. Hamilton M. A rating scale for depression. J Neurol Neurosurg Psychiatry 1960; 23:56–62IDS-C

2. Rush, A.J., et al., The Inventory for Depressive Symptomatology (IDS): preliminary findings. Psychiatry Res, 1986. 18(1): 65-87.

3. Rush AJ, Trivedi MH, Ibrahim HM, Carmody TJ, Arnow B, Klein DN, et al. The 16-Item Quick Inventory of Depressive Symptomatology (QIDS), clinician rating (QIDS-C), and self-report (QIDS-SR): A psychometric evaluation in patients with chronic major depression. Biol Psychiatry. 2003;54(5):573-83.

4. Gibbons RD, Hooker G, Finkelman MD, Weiss DJ, Pilkonis PA, Frank E, et al. (2013): The computerized adaptive diagnostic test for major depressive disorder (CAD-MDD): a screening tool for depression. J Clin Psychiatry. 74:669-674.

5. Gibbons RD, Weiss DJ, Kupfer DJ, Frank E, Fagiolini A, Grochocinski VJ, et al. (2008): Using computerized adaptive testing to reduce the burden of mental health assessment. Psychiatr Serv. 59:361-368.

6. Gibbons RD, Weiss DJ, Pilkonis PA, Frank E, Moore T, Kim JB et al. (2012): Development of a computerized adaptive test for depression. Arch Gen Psychiatry. 69: 1104-1112.

7. Nolen-Hoeksema, S. The role of rumination in depressive disorders and mixed anxiety/depressive symptoms. J Abnorm Psychol, 2000. 109(3): 504-11.

8. Wells, A. and M.I. Davies, The Thought Control Questionnaire: A measure of individual differences in the control of unwanted thoughts. Behav Res Ther, 1994. 32(8): 871-8.

9. Henry, J.D. and J.R. Crawford, The short-form version of the Depression Anxiety Stress Scales (DASS-21): construct validity and normative data in a large non-clinical sample. Br J Clin Psychol, 2005. 44(Pt 2): 227-39.

10. Osman, A., et al., The Depression Anxiety Stress Scales-21 (DASS-21): further examination of dimensions, scale reliability, and correlates. J Clin Psychol, 2012. 68(12): 1322-38.

11. Young, R.C., et al., A rating scale for mania: reliability, validity and sensitivity. Br J Psychiatry, 1978. 133: 429-35.

12. Williams JM, Broadbent K. Autobiographical memory in suicide attempters. Journal of abnormal psychology. 1986;95(2):144-9.

13. Conway CC, Slavich GM, Hammen C. Dysfunctional Attitudes and Affective Responses to Daily Stressors: Separating Cognitive, Genetic, and Clinical Influences on Stress Reactivity. Cognit Ther Res. 2015;39(3):366-77. PMCID: PMC4817852

images within each subject, the minimal preprocessing pipelines available through the HCP project will be used to segment DLPFC and dACC subregions using the Desikan Atlas. Linear regression analyses will then determine relationships between 1) mean magnetic field and CBF at baseline and change in clinical and cognitive scores across the tDCS trial and 2) mean magnetic field and change in rCBF across the treatment trial within DLPFC and dACC ROIs [**Fig 10** shows correlations between tDCS current intensity and rCBF increase]. For predictive models using ROIs, FDR thresholding at .05 will control for the multiple outcomes (mood scores and measures of cognitive control/response inhibition and emotional bias).

Though not an aim of this proposal, sex effects and interactions with sex will be examined in follow-up analyses.

In post-hoc analysis, structural image analysis will also allow us to determine whether variations in *brain morphometry* interact with target engagement. Further, functional connectivity (FC) analysis will be applied on ASL perfusion images[127] to detect potential changes in FC of resting networks using ROIs or ICA.

C8. Power: Our earlier work indicates that a sample size of n=20 can reliably detect a 15% change in rCBF across time using 3D GRASE pCASL[126] [**Fig 11**]. A minimum signal change of 15% will thus define *within-group* modulation of the DLPFC and dACC with active tDCS. With n=20 subjects in each condition (C-tDCS, HD-tDCS and Sham), we will have 80% power to detect changes in rCBF *between groups* with an effect size of 0.91 and a two-tailed *p=0.05, assuming a two sample t-test comparing pairs of groups or an effect of 0.78 for the active (n=40) vs. sham (n=20) arms.* The t-test approximates the power for the assessment of the group by time interaction term from the mixed model

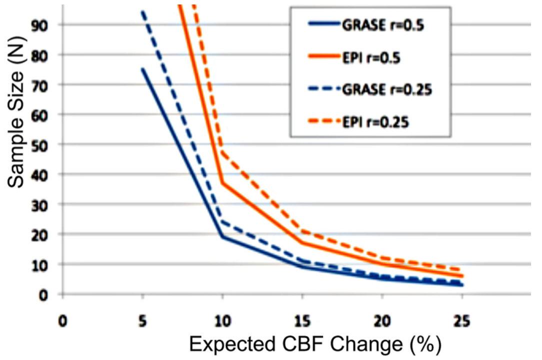

Fig 11. Estimated sample size for a within-subject design with one baseline and follow-up measurement for GRASE and EPI pCASL respectively, with an assumed correlation coefficient of 0.25–0.5 between repeated measures (two-sided α=.05; power=90%).

A meta-analysis evaluating the antidepressant effects of left anodal DLPFC tDCS including 176 patients revealed significant effects for active versus sham tDCS on symptoms examined as a continuous measure (p=.006)[3]. The pooled estimate of effect size (*Hedges' g*) was 0.74 (95% confidence interval (CI) 0.21–1.27). Though a more recent meta-analysis including only RCTs (259 patients) produced smaller effects size estimates (*g*=.40, 95%CI 0.07–0.73)[2]. Notably, trials included in both meta-analyses differed with regard to stimulation intensity, trial length and clinical variables and included relatively small samples. Thus, the sample size for the R33 phase is based on an effect size estimate intermediate to the pooled effect sizes reported across these meta-analyses, which is 0.57. Using an effect size of 0.57, we will have 80% power to detect significant *between group* changes in tDCS-related change in depressive symptoms with α=.05 (two-tailed) and with n=50 in each tDCS condition. This assumes a two sample t-test comparing change scores between groups, which is a simplification of the repeated measures analysis plan. With n=50 in the active tDCS group, we will have 80% power to detect a correlation (Pearson) between change in CBF vs. clinical or behavioral scores of r=0.38 with a two sided α=.05. The effect size estimated to determine sample size may be slightly conservative since: a) the current study is expected to gain power by optimizing target engagement in the R61 phase, b) earlier results suggest that longer tDCS trials (>10 days)[2] and c) the use of tDCS as an adjunct to standard antidepressant treatment may enhance effects.

***Rigor and reproducibility** is addressed in the Approach a described in the sections above, including using a) a randomized double-blind design for both project phases (see Other Attachment for details of blinding procedures and other methods to reduce bias), b) improving precision and reducing variability in measurements by controlling for variables such as age, medication status, BMI, the timing and location of procedures and including MRI-guided methods to enhance precision of electrode placement c) including a sufficiently powered and detailed statistical plan with strategies for handling missing data.*

C9. Timeline: Our timeline and study milestones are detailed and illustrated in the Other Attachment. In brief, following project set-up occurring during the first 2-months of the funding period, recruitment, screening and data collection and analysis will occur between month 3 and 18 for the R61. Analyses to determine go-no-go criteria will occur at month 23. For the R33, subject enrollment and testing will occur during months 3-30, with final analysis occurring between months 30-36. In the R61 phase, we expect to enroll 2-3 subjects a month (32-33 monthly tDCS sessions). In the R33 phase, we again expect to enroll 2-3 subjects a month (33-34 monthly tDCS sessions).
