# Supplementary material for: Personalized High-Definition Transcranial Direct Current Stimulation for the Treatment of Depression: A Randomized Clinical Trial
Source: JAMA Netw Open. 2025 Sep 11;8(9):e2531189. doi: 10.1001/jamanetworkopen.2025.31189 (PMC12426800; doi:10.1001/jamanetworkopen.2025.31189)
Supplement: Supplement 2. — eMethods 1. Inclusion and Exclusion Criteria eMethods 2. Trial Dropouts eMethods 3. Personalization Methodology eMethods 4. HAMD Scores at Consultation and Baseline eResults 1. Treatment-Related Discomfort eResults 2. Treatment-Related Changes in HAMD Symptom Dimensions eResults 3. Treatment-Related Changes in Individual HAMD Items eFigure. Spaghetti Plots of HAMD Scores Over Time eTable. Outcome Measures Over Time eReferences [file jamanetwopen-e2531189-s002.pdf]

Supplementary Online Content

Jog MA, Norris V, Pfeiffer P, et al. Personalized high-definition transcranial direct current stimulation for the treatment of depression: a randomized clinical trial. *JAMA Netw Open.* 2025;8(9):e2531189. doi:10.1001/jamanetworkopen.2025.31189

eMethods 1. Inclusion and Exclusion Criteria
eMethods 2. Trial Dropouts
eMethods 3. Personalization Methodology
eMethods 4. HAMD Scores at Consultation and Baseline
eResults 1. Treatment-Related Discomfort
eResults 2. Treatment-Related Changes in HAMD Symptom Dimensions
eResults 3. Treatment-Related Changes in Individual HAMD Items
eFigure. Spaghetti Plots of HAMD Scores Over Time
eTable. Outcome Measures Over Time
eReferences

This supplementary material has been provided by the authors to give readers additional information about their work.

© 2025 Jog MA et al. *JAMA Network Open*.

eMethods 1. Inclusion and Exclusion Criteria

For inclusion, participants needed to meet criteria for a current major depressive episode, assessed using the Mini International Neuropsychiatric Interview (MINI[1]) 7.0.2 for DSM-V. The MINI was also used to exclude other diagnoses specified below under "Exclusion Criteria". Additional criteria are listed below:

Inclusion criteria:

1. Age between 18 to 65 years, inclusive
2. Capacity to provide informed consent
3. Hamilton Rating Scale for Depression (HAMD[2]) score ≥14 and <24, with or without symptoms of anxiety.
4. Treatment naïve or on a stable standard antidepressant regimen (including selective serotonin reuptake inhibitors (SSRIs), serotonin-noradrenaline reuptake inhibitors (SNRIs), monoamine oxidase inhibitors (MOAIs) or tricyclic's (TCAs)) with no change in treatment 6-weeks prior to and during the transcranial direct current stimulation (tDCS) intervention.
5. Within traveling distance of the University of California Los Angeles (UCLA)

Exclusion criteria:

1. Pregnancy (to adhere to the policies of the UCLA Brain Mapping Center and because this is a research study and other treatment options are available)
2. Non-English speaking (due to scales administered)
3. Substance Use Disorder within the last 3 months
4. Neurological condition associated with brain abnormalities (e.g., traumatic brain injury; recent stroke, tumor)
5. Any contraindication to tDCS (e.g., skin disease or treatment causing irritation)
6. Any condition that would contraindicate MRI (metal implants, claustrophobia or a breathing or movement disorder)
7. Currently receiving any form of Cognitive Behavioral Therapy, Dialectical Behavioral Therapy, or Acceptance and Commitment Therapy
8. Change in antidepressant medication within 6-weeks of starting the trial
9. Severe or treatment resistant depression – HAMD scores > 24 and a history of a major depressive episode lasting >2-years or failure to 2 or more antidepressant trials in the current index episode
10. Any neuromodulation therapy (e.g., ECT, rTMS, DBS, VNS) within the last 3-months
11. Past tDCS treatment
12. Current or past (within the last 1-month) use of anticonvulsants, lithium, dexamphetamine
13. Current use of decongestants or other medication including sleeping aids previously shown to interfere with cortical excitability
14. Diagnosis of Schizophrenia Axis I disorder, or dementia of any type
15. Diagnosis of Bipolar I disorder (due to possible risk of mania and because lithium and anticonvulsants are excluded).
16. Diagnosis of primary anxiety disorder
17. Diagnosis of seizure disorder or history of seizures
18. Depression related to serious medical illness (i.e., mood disorder due to general medical condition)
19. Actively suicidal as defined by a score of 4 on item 3 of HAMD
20. Unavailable/predicted to miss more than 2 study visits
21. Medical conditions that render the participant high-risk for COVID-19 infection (e.g. diabetes, obesity).
22. Substantial changes to hair styles that may impact the fit of the personalized electrode cap for the duration of the trial (See S4 for details on the personalization procedure)

© 2025 Jog MA et al. *JAMA Network Open*.

eMethods 2. Trial Dropouts
N=71 participants were enrolled in the study and randomized to receive active (N=40) or sham (N=31) tDCS treatment. Of these, (a) one participant in the sham group requested to reschedule the MRI conducted during the first treatment session and was subsequently lost to follow-up, (b) one participant in the active group communicated feeling depressed and irritable during the first session and requested to discontinue participation, and (c) one participant randomized to the active group had pre-existing scalp dermatitis that was aggravated with tDCS. No treatment-related data had been acquired for (a) and (b) before subject dropout. Regarding (c), the subject and investigators agreed that study withdrawal was necessary; the skin condition is a contraindication[3] that all participants are asked about during the initial phone screening step, and is specified as exclusionary in the telephone screening script. The remaining 68 participants completed all study activities and follow-ups, and did not have instances of missing data.

eMethods 3. Personalization Methodology
The High-Definition tDCS (HD-tDCS) configuration was personalized to target a specific coordinate in the left dorsolateral prefrontal cortex (DLPFC) in each participant ([x=−46, y=44, z=38] mm MNI coordinate space). Personalization was carried out as follows: first, the location of this coordinate in individual participants was determined by processing their structural MRI data (acquired during the consult study-visit) using inverse-normalization in SPM12[4]. Next, frameless stereotaxic neuronavigation (Brainsight[5]) was used to mark the scalp projection of this coordinate onto caps[6] that were individually fitted to study participants. These personalized caps were then used to securely place electrodes for all subsequent HD-tDCS treatment sessions. For additional methodological details, see [7]. Note that the procedures used in all of the above steps – coordinate localization, personalized cap fitting and electrode placement are robust, and have been validated for HD-tDCS applications[7].

Recent work[7] employing HD-tDCS electric field (EF) simulations indicates that the personalization methodology described above more reliably targets the specified anatomical coordinate in the left DLPFC. Briefly, in the absence of personalization, one is limited to using scalp landmarks. This can result in imprecise electrode placement over the left DLPFC brain-target, with placement inaccuracies on the order of 2cm (due to the imprecise cortico-scalp relationship and operator errors)[7]. 2cm inaccuracies are predicted to induce large-scale deviations in the HD-tDCS EF distribution near the left DLPFC target, as shown quantitatively in the paper[7].

In this study, placement inaccuracies were measured at the mid-treatment timepoint. Here, after study staff had determined the locations for placing the HD-tDCS electrodes using the individually-fitted caps and procedures described above, neuronavigation equipment was used to record the position of these locations relative to the intended target. Placement inaccuracies were measured to be (Mean±SD (Standard deviation)) 4.25±2.81 mm in the active group and 4.19±2.07 mm in the sham group. Notably, average inaccuracies in both groups were less than 1 cm (i.e., 10 mm), for which simulations predict minimal changes in the EF distribution near the left DLPFC target[7]. Placement inaccuracies also did not significantly differ between the two treatment groups (p=0.92, 2-sample t-test).

eMethods 4. HAMD Scores at Consultation and Baseline
Participants were evaluated for eligibility during the consult study visit. Upon enrollment, the 12-day HD-tDCS therapy typically started after 2 weeks due to the need to acquire structural MRI data (for personalizing

© 2025 Jog MA et al. *JAMA Network Open*.

treatments, see **eMethods 3**), and accommodate preferences in scheduling the 12 consecutive working day in-person treatment visits. HAMD scores were remeasured during the first treatment visit before the administration of HD-tDCS. Group differences in scores between these two timepoints (i.e. consult and baseline) were investigated using 2-sample t-tests.

No significant group differences were observed at either timepoint (<u>Consult</u>: p=0.80, HAMD scores (mean±SD) in [active-HD]:18.2±2.8, [sham-HD]:18.0±2.9; <u>Baseline</u>: p=0.50, HAMD scores in [active-HD]:17.2±3.2, [sham-HD]:17.7±3.3). Group differences in the HAMD score change over this duration were also minimal and non-significant (p=0.36, changes in HAMD scores in [active-HD]:-1.0±3.3, [sham-HD]:-0.3±3.0 points).

eResults 1. Treatment-Related Discomfort

Treatment related discomfort was assessed (a) immediately after each HD-tDCS treatment using the Generic Assessment of Side Effects (GASE[8]) scale, and (b) at the post-treatment time-point using the Adverse Events Questionnaire (AEQ[9]). The GASE scale measured acute side-effects of the administered treatment, while the AEQ measured side-effects manifesting over the course of the 12-day therapy.

GASE data were averaged over the 12 treatment timepoints and two-sample t-tests were used to evaluate group differences between overall scores as well as individual item scores. Group differences in the overall as well as individual AEQ item scores were also evaluated using two-sample t-tests.

The overall GASE and AEQ scores did not differ significantly between the active and sham treatment groups (p_{GASE}=0.62; p_{AEQ}=0.52). Some individual item scores showed significant group differences (<u>GASE-items</u>: Skin Rash or Itching, **eResults 1.A**; <u>AEQ-items</u>: Burning sensation and Skin redness, **eResults 1.B**); however, the average group difference between the active and sham groups in all of these cases was <0.3 points on the 4 point Likert scale (0,1,2,3 = absent, mild, moderate and severe levels of discomfort respectively). The overall prevalence of each side-effect item is also shown in **eResults 1.A** and **eResults 1.B**; acute skin rash or itching was reported by 5.8% of the study sample, while burning sensation and skin redness was reported by less than 15% of study participants.

eResults 1.A: Treatment related acute side-effects (Generic Assessment of Side Effects (GASE) scale).

#	GASE symptoms (%[a])	p-value (*: p<0.05)	Mean Group difference	active		sham	
				Mean	SD	Mean	SD
1	Headache (1.5%)	0.44	0.045	0.145	0.284	0.1	0.164
2	Dizziness (0%)	0.096	-0.05	0.028	0.068	0.082	0.185
3	Palpitations, irregular heartbeat (0%)	0.775	-0	0.009	0.053	0.012	0.039
4	Breathing problems (0%)	0.423	-0.01	0.002	0.013	0.008	0.046
5	Nausea (0%)	0.29	-0.02	0.006	0.03	0.031	0.138
6	Skin Rash or Itching (5.8%)	**0.024***	0.169	0.184	0.397	0.014	0.063
7	Fever, increased temperature (0%)	0.257	-0	0	0	0.003	0.015
8	Fatigue, loss of energy (2.9%)	0.469	-0.05	0.106	0.303	0.153	0.205

[a]: % of subjects in the total sample who reported experiencing at least mild symptoms due to HD-tDCS therapy.

eResults 1.B: Side-effects after 12 days of HD-tDCS therapy (Adverse Events Questionnaire (AEQ))

#		p-value		active	Sham

© 2025 Jog MA et al. *JAMA Network Open*.

	AEQ symptoms (%[a])	(*: p<0.05)	Mean Group difference	Mean	SD	Mean	SD
1	Headache (8.8%)	0.504	0.075	1.25	0.5	1.175	0.395
2	Neck pain (2.9%)	0.687	-0.019	1.039	0.179	1.058	0.204
3	Scalp pain (16.2%)	0.068	0.216	1.316	0.574	1.1	0.305
4	Tingling (13.2%)	0.978	-0.003	1.23	0.463	1.233	0.464
5	Itching (4.4%)	0.415	-0.063	1.079	0.273	1.142	0.358
6	Burning sensation (14.7%)	**0.03***	0.246	1.329	0.507	1.083	0.373
7	Skin redness (13.2%)	**0.006***	0.274	1.316	0.509	1.042	0.162
8	Sleepiness (19.1%)	0.962	-0.006	1.303	0.476	1.308	0.503
9	Trouble concentrating (7.4%)	0.492	-0.063	1.112	0.264	1.175	0.479
10	Acute mood change (7.4%)	0.613	0.038	1.171	0.291	1.133	0.32
11	Others (3.0%)	0.167	-0.14	1.026	0.113	1.167	0.606

[a]: % of subjects in the total sample who reported experiencing at least mild symptoms due to HD-tDCS therapy.

eResults 2. Treatment-Related Changes in HAMD Symptom Dimensions

Symptom dimensions within the HAMD have been identified by factor analyses[10]. These dimensions include anxiety, depression, somatic and insomnia symptoms, and were determined by the data-driven factor analysis using datasets that were independent of the current study.

We used 2-sample t-test to explore whether pre- to post-treatment changes in symptom dimensions differed between the active and sham treatment groups. As shown in **eResults 2.1**, pre- to post-treatment changes in the anxiety symptom dimension differed significantly between the two treatment groups (group-difference=-0.68±1.42, *p*=0.049, *d*=-0.48(-0.96,-0.00)). Post-hoc t-tests showed significant decreases over time within both groups, with (significantly) greater decreases in the active treatment group (changes over time in [active-HD]: -1.6±1.4, [sham-HD]: -1.0±1.4). Follow-up t-tests revealed that this improvement was driven by significant active treatment-related improvements in the psychic and somatic anxiety items constituting the anxiety symptom cluster (psychic anxiety: *p*=0.030, *d*=-0.54(-1.02,-0.05); somatic anxiety: *p*=0.047, *d*=-0.49(-0.98,0)). Group differences in the remainder of the symptom clusters were not statistically significant.

© 2025 Jog MA et al. *JAMA Network Open*.

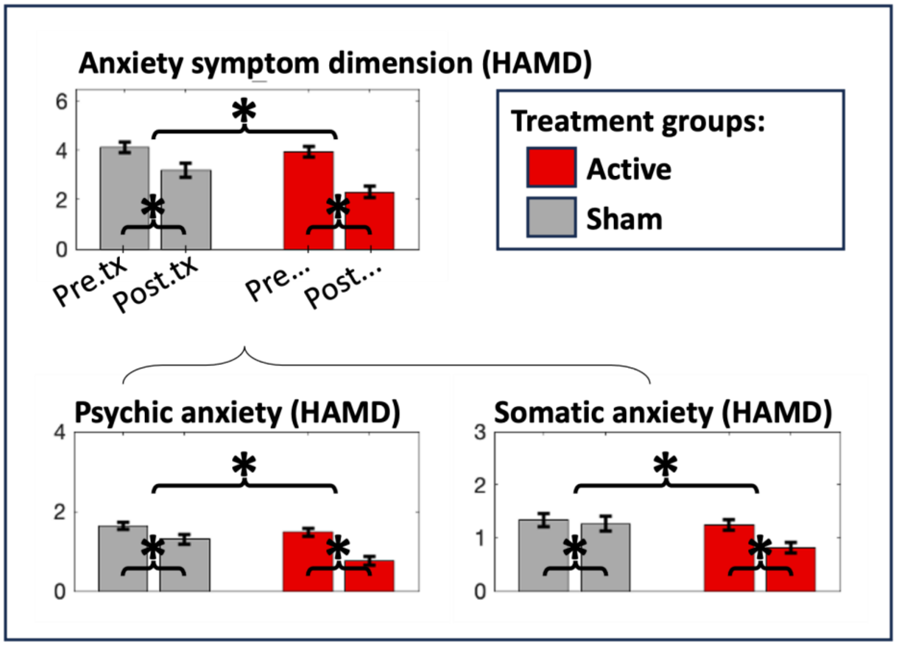

eResults 2.1: Treatment-related changes in the anxiety symptom dimension within the HAMD. The top row shows the anxiety symptom dimension scores over time and across treatment groups. Scores are plotted on the y-axis and timepoints on the x-axis; 'Pre.tx' and 'Post.tx' refer to pre- and post-treatment timepoints respectively. Significant ($p<0.05$) group differences in the pre- to post-treatment score change were observed. The bottom row shows the constituent items of the anxiety symptom cluster where significant group differences in score changes were also observed. In all cases, post-hoc t-tests showed greater score decreases, i.e. superior improvement, in the active treatment group. Error-bars in the plot reflect standard error. * highlights significant ($p<0.05$) results.

eResults 3. Treatment-Related Changes in Individual HAMD Items

The HAMD measure used in this trial represents an overall measure of depression severity and was comprehensively investigated in the analyses described in the main manuscript. Each final HAMD score is obtained by summing over 17 different scored items. Treatment-related worsening of individual items (e.g. suicidality) is salient in determining safety. Consequently, we explored whether any individual item-scores worsened due to treatment.

One-sample t-tests were used to evaluate pre- to post-treatment item-score changes within each treatment group. As shown in **eResults 3.1**, significant differences (when observed) arose due to significant score decreases, i.e., symptom improvements. Notably, symptom improvements were more prevalent in the active treatment group than the sham group (16 vs. 9 for the active and sham groups respectively).

Group differences in the pre- to post-treatment score changes were also evaluated (using 2-sample t-tests). Score changes significantly differed between the treatment groups for the psychic and somatic anxiety items. In both cases, scores were observed to significantly improve with active treatment compared to sham, consistent with the results observed in the main manuscript (Results section, Exploratory analyses sub-section).

© 2025 Jog MA et al. *JAMA Network Open*.

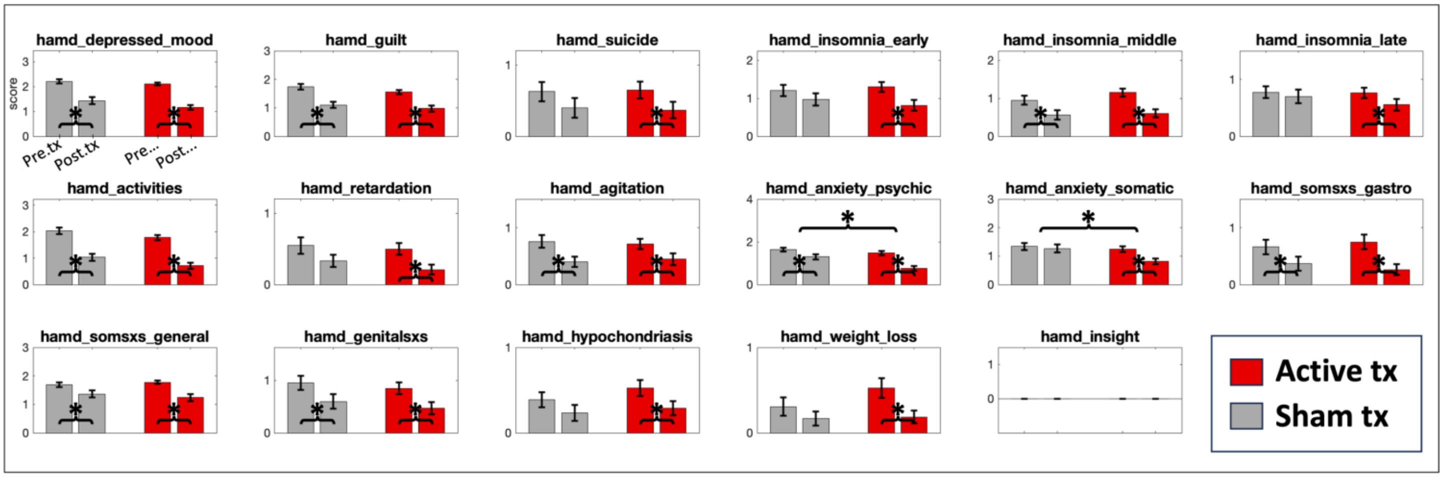

eResults 3.1: Treatment-related changes in individual HAMD items. Each HAMD item is plotted in a separate panel, with item-scores on the y-axis and time and treatment groups on the x-axis. 'Pre.tx' and 'Post.tx' refer to pre-treatment and post-treatment timepoints respectively. Significant (p<0.05) differences are highlighted with a '*'. The barplot for the insight item (bottom right) looks markedly different because most subjects showed minimal pre-treatment severity and were scored '0'. Treatment-related changes in this item were also minimal and non-significant.

eFigure. Spaghetti Plots of HAMD Scores Over Time

The left and right subplots show the 17-item Hamilton Depression Rating Scale (HAMD) scores over time for each participant in the active and sham treatment groups respectively. HAMD scores are plotted on the y-axis and timepoints are shown on the x-axis, with pre.tx, mid.tx, post.tx, +2wk and +4wk indicating the pre-, mid-, post-, 2 weeks post- and 4 weeks post-treatment timepoints respectively. Note that the mid- and post-treatment timepoints correspond to treatment-visits #6 and #12 of HD-tDCS therapy administered over 12 consecutive working days. The HAMD scores reflect depression severity, and a decrease in the HAMD scores over time indicates mood improvement.

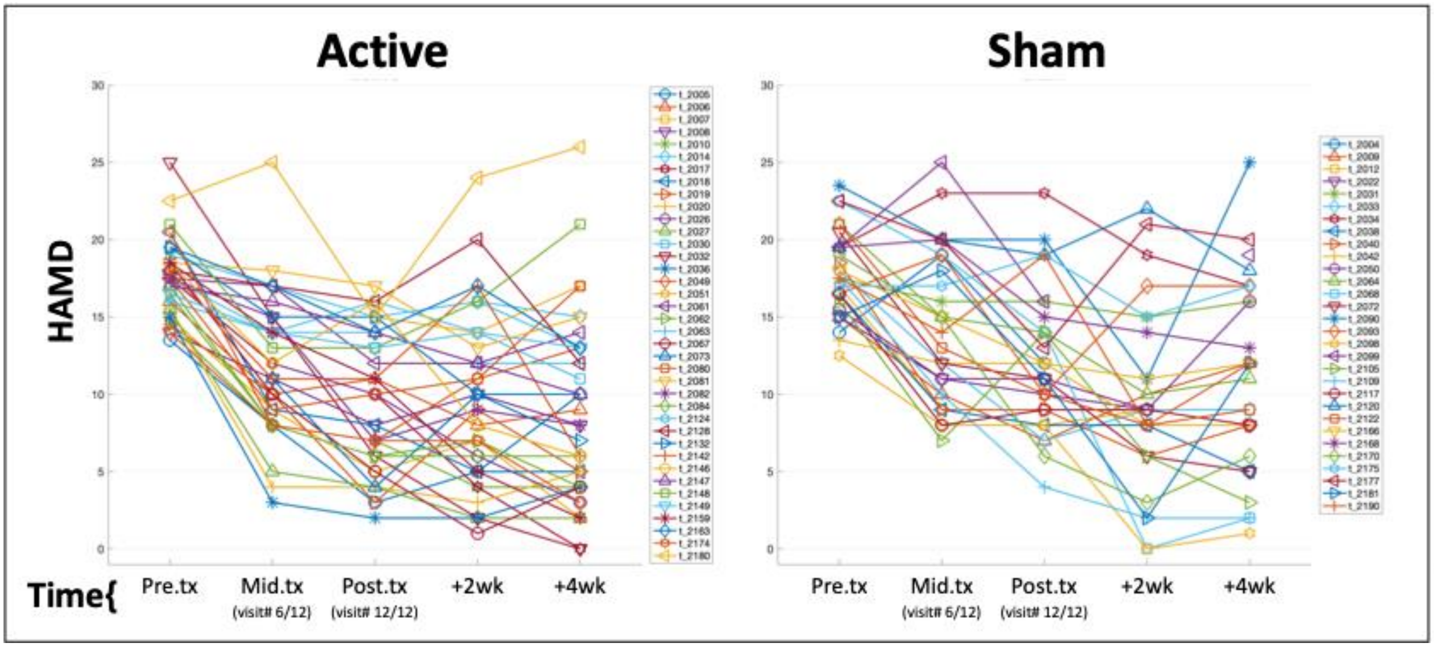

© 2025 Jog MA et al. *JAMA Network Open*.

eTable. Outcome Measures Over Time

Outcome measures	Group[a]	TimePoints[b]			
		Mid.tx	Post.tx	+2wk	+4wk
**Primary**					
Change in HAMD[c] (relative to Pre.tx[b])	Active	-5.49 ± 3.72	-7.75 ± 4.21	-7.91 ± 5.37	-8.80 ± 5.95
	Sham	-3.30 ± 4.31	-5.60 ± 4.37	-8.29 ± 5.6	-7.00 ± 5.61
**Secondary**					
%ch in HAMD[c] (relative to Pre.tx[b])	Active	-31.9 ± 21.41	-44.6 ± 23.9	-45.1 ± 28.32	-50 ± 30.71
	Sham	-18.6 ± 25.05	-31.3 ± 23.54	-46.9 ± 29.66	-39.9 ± 30.7
Responders	Active	7	16	17	19
	Sham	3	8	14	12
Remitters	Active	3	15	15	18
	Sham	1	4	8	8

[a]: N=38(active), 30(sham).

[b]: Pre.tx, Mid.tx, Post.tx, +2wk and +4wk correspond to the pre-, mid-, post-,2 weeks post- and 4 weeks post-treatment timepoints respectively. Here, the mid- and post-treatment timepoints correspond to treatment-visits #6 and #12 of HD-tDCS therapy administered over 12 consecutive working days.

[c]: are reported as Mean ± Standard deviation

© 2025 Jog MA et al. *JAMA Network Open*.

eReferences

1. Sheehan DV, Lecrubier Y, Sheehan KH, et al. The Mini-International Neuropsychiatric Interview (M.I.N.I.): the development and validation of a structured diagnostic psychiatric interview for DSM-IV and ICD-10. *J Clin Psychiatry*. 1998;59 Suppl 20:22-33;quiz 34-57.
2. Hamilton M. A rating scale for depression. *J Neurol Neurosurg Psychiatry*. Feb 1960;23:56-62. doi:10.1136/jnnp.23.1.56
3. Thair H, Holloway AL, Newport R, Smith AD. Transcranial Direct Current Stimulation (tDCS): A Beginner's Guide for Design and Implementation. *Front Neurosci*. 2017;11:641. doi:10.3389/fnins.2017.00641
4. Penny WD, Ashburner J, Kiebel S, et al. Statistical parametric mapping: An annotated bibliography. http://www.fil.ion.ucl.ac.uk/spm/bib.htm
5. Brainbox-Neuro. Brainsight Neuronavigation. Accessed 23 Dec, 2019. https://brainbox-neuro.com/catalogue/neuro-navigation/tms-navigation/brainsight-tms-navigation/
6. EASYCAP GmbH. Landmark Caps (Product ID: LM-UCHW-xx). Accessed 15 Feb, 2025. https://www.easycap.de/
7. Jog M, Anderson C, Kim E, et al. A novel technique for accurate electrode placement over cortical targets for transcranial electrical stimulation (tES) clinical trials. *J Neural Eng*. Oct 11 2021;18(5)doi:10.1088/1741-2552/ac297d
8. Rief W, Barsky AJ, Glombiewski JA, Nestoriuc Y, Glaesmer H, Braehler E. Assessing general side effects in clinical trials: reference data from the general population. *Pharmacoepidemiol Drug Saf*. Apr 2011;20(4):405-15. doi:10.1002/pds.2067
9. Brunoni AR, Amadera J, Berbel B, Volz MS, Rizzerio BG, Fregni F. A systematic review on reporting and assessment of adverse effects associated with transcranial direct current stimulation. *Int J Neuropsychopharmacol*. Sep 2011;14(8):1133-45. doi:10.1017/S1461145710001690
10. Shafer AB. Meta-analysis of the factor structures of four depression questionnaires: Beck, CES-D, Hamilton, and Zung. *J Clin Psychol*. Jan 2006;62(1):123-46. doi:10.1002/jclp.20213

© 2025 Jog MA et al. *JAMA Network Open*.
