# Supplementary material for: Personalized High-Definition Transcranial Direct Current Stimulation for the Treatment of Depression: A Randomized Clinical Trial
Source: JAMA Netw Open. 2025 Sep 11;8(9):e2531189. doi: 10.1001/jamanetworkopen.2025.31189 (PMC12426800; doi:10.1001/jamanetworkopen.2025.31189)
Supplement: Supplement 3. — Data Sharing Statement [file jamanetwopen-e2531189-s003.pdf]

Data Sharing Statement

Jog. Personalized High-Definition Transcranial Direct Current Stimulation for the Treatment of Depression. *JAMA Netw Open*. Published September 11, 2025.
doi:10.1001/jamanetworkopen.2025.31189

Data
Additional Information: Trial registry: clinicaltrials Registry URL: https://clinicaltrials.gov/ Trial registration number: NCT04507243
Data available: Yes
Data types: Deidentified participant data
How to access data: The data is available on the NIMH data archive (https://nda.nih.gov/edit_collection.html?id=2737)
When available: With publication

Supporting Documents
Document types: None

Additional Information
Who can access the data: The data is available on the NIMH data archive (https://nda.nih.gov/edit_collection.html?id=2737)
Types of analyses: For any purpose
Mechanisms of data availability: To request access of the data, researchers will use the standard processes at NDA, and the NDA Data Access Committee will decide which requests to grant. The standard NDA data access process allows access for one year and is renewable.
